# PPARα Deficiency in Inflammatory Cells Suppresses Tumor Growth

**DOI:** 10.1371/journal.pone.0000260

**Published:** 2007-02-28

**Authors:** Arja Kaipainen, Mark W. Kieran, Sui Huang, Catherine Butterfield, Diane Bielenberg, Gustavo Mostoslavsky, Richard Mulligan, Judah Folkman, Dipak Panigrahy

**Affiliations:** 1 Vascular Biology Program, Department of Surgery, Children's Hospital, Harvard Medical School, Boston, Massachusetts, United States of America; 2 Department of Pediatric Oncology, Dana-Farber Cancer Institute, Harvard Medical School, Boston, Massachusetts, United States of America; 3 Department of Genetics, Harvard Medical School, Boston, Massachusetts, United States of America; Ordway Research Institute, Inc., United States of America

## Abstract

Inflammation in the tumor bed can either promote or inhibit tumor growth. Peroxisome proliferator-activated receptor (PPAR)α is a central transcriptional suppressor of inflammation, and may therefore modulate tumor growth. Here we show that PPARα deficiency in the host leads to overt inflammation that suppresses angiogenesis via excess production of the endogenous angiogenesis inhibitor thrombospondin-1 and prevents tumor growth. Bone marrow transplantation and granulocyte depletion show that PPARα expressing granulocytes are necessary for tumor growth. Neutralization of thrombospondin-1 restores tumor growth in PPARα-deficient mice. These findings suggest that the absence of PPARα activity renders inflammatory infiltrates tumor suppressive and, thus, may provide a target for inhibiting tumor growth by modulating stromal processes, such as angiogenesis.

## Introduction

Non-neoplastic “host” cells, such as endothelial, stromal and inflammatory cells, play a critical role in tumor growth; and genes prognostic for cancer outcome may be expressed in the non-neoplastic tissue compartment [1]. While tumor angiogenesis has been intensely studied for more than two decades and has become an accepted target in cancer therapy, it is only in the last few years that inflammation has entered center stage of investigations into non-cell autonomous processes in cancer.

Chronic inflammation in the tumor stroma has long been known to contribute to tumor progression. Increased infiltration of innate immune cells to the tumor, such as macrophages, mast cells and neutrophils, correlates with increased angiogenesis and poor prognosis [2], [3]. In contrast, lymphocytic/monocytic inflammatory infiltrates are sometimes associated with tumor inhibition and a more favorable prognosis [3]–[5]. Recently, NF-κB, a central positive regulator of inflammation, has emerged as a molecular link between inflammation and cancer growth. NF-κB promotes tumor growth not only in a cancer cell-autonomous manner by transactivating anti-apoptotic genes, but it also stimulates inflammatory processes in the microenvironment that lead to the production of tumor-promoting cytokines [6].

Conversely, PPARα, a ligand-activated nuclear receptor/transcription factor, is a key negative regulator of inflammation. Activation of PPARα by ligands inhibits inflammation [7] whereas PPARα deficient mice exhibit enhanced inflammation [8]. Despite PPARα's role in suppressing inflammation, it appears to be necessary and sufficient for rodent tumorigenesis [9]. In fact, prolonged PPARα activation by peroxisome proliferators induces hepatocarcinogenesis in rodents; conversely PPARα KO mice are resistant to tumorigenesis induced by PPARα agonists [10], [11]. This may be due in part to cell-autonomous effect of PPARα, because it is expressed in many tumor cell lines [12], [13]. Another possibility is that in PPARα deficient mice, stromal processes, such as inflammation, inhibit tumor growth, which results in microscopic-sized tumors that remain dormant. The role of PPARα in inflammation has been extensively studied in normal physiological processes (wound healing) and cardiovascular diseases (atherosclerosis) [14], [15]; but the effect of PPARα mediated suppression of inflammation on tumors has not been characterized. Here we show that overt inflammation in the absence of PPARα in the host tissue prevents tumor growth. This indicates that in contrast to the emerging notion that inflammatory infiltrates promote tumors, the specific nature of the inflammatory process must be considered when linking inflammation to tumorigenesis.

## Results

### Deletion of PPARα in Host Tissue inhibits Tumor Growth and Metastasis

**Figure 1:**
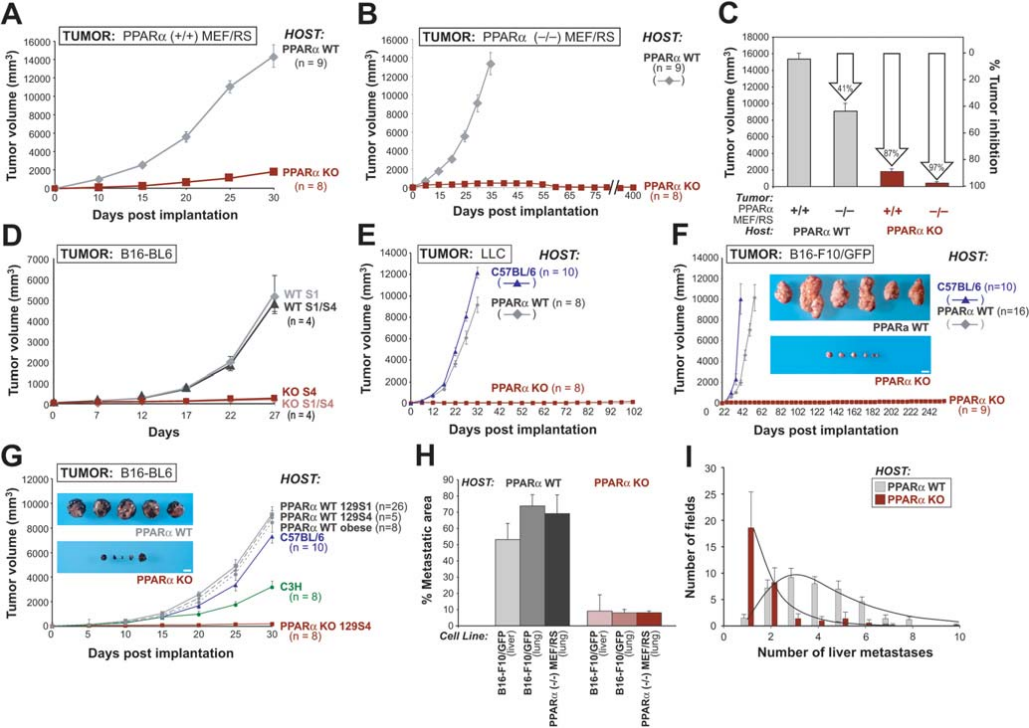
Tumor growth and metastasis are inhibited in PPARα knockout (KO) mice. PPARα wild type (WT) and PPARα KO mice were injected subcutaneously or intravenously with various tumor cell lines; (n) = number of mice/group. (A–C) The growth of engineered PPARα(+/+) MEF/RS and PPARα(−/−) MEF/RS tumors in PPARα WT and KO mice. (A) PPARα(+/+)MEF/RS tumor growth in PPARα WT (♦ gray) and KO (▪ brown) mice. (B) The growth curves of PPARα(−/−)MEF/RS in PPARα WT (♦ gray) and KO (▪ brown) mice.(C) Columns summarize the inhibitory effect of PPARα (−/−) tumor and host cells at day 30 post implantation (average±standard error of the mean). (D–F) The growth of different murine tumors in different mouse strains. (D) The growth of B16-BL6 melanoma was compared in WTS1 (♦ gray), WTS1/S4 (▴gray), KOS4 (▪ brown) and KO S1/S4 (♦ pink) strains. WT S1/S4, PPARα WT second generation littermates from PPARα WT 129/S1 and KO 129/S4; KO S1/S4, PPARα KO second generation littermates from PPARα KO 129/S4. (E) Lewis lung carcinoma growth in PPARα WT (♦ gray), PPARα KO (▪ brown) and C57BL/6 (▴ blue) mice. (F) B16-F10/GFP tumor growth in PPARα WT, PPARα KO and C57BL/6 mice, blue insets demonstrate representative B16-F10/GFP tumors in PPARα WT and KO mice on day 30 post implantation. Scale bar, 1 cm. (G) B16-BL6 melanoma was implanted in mice of indicated genetic backgrounds. Representative B16-BL6 tumors in PPARα WT (♦ gray) and PPARα KO (▪ brown) mice on day 30 post implantation are shown (blue insets). Scale bar, 1 cm. (H–I) Metastasis in PPARα WT and KO mice. H: Metastatic areas of B16-F10/GFP and PPARα(−/−)MEF/RS tumor cells at day 21 post-injection in lung and liver of PPARα WT (♦ gray) and KO mice (▪ brown). I: Number of liver metastases in PPARα WT (▪ gray) and KO (▪ brown) mice injected with B16-F10/GFP tumor cells (average±standard deviation).

We used several murine models to determine how the increased inflammatory response observed in the absence of PPARα affects tumor growth and metastasis. Fi rst, we stably transformed mouse embryonic fibroblasts (MEF) with SV40 large T antigen and H-ras [16] to obtain isogeneic tumorigenic cell lines that were either wild type (PPARα(+/+)MEF/RS) or lacked PPARα (PPARα(−/−)MEF/RS). These two tumorigenic cell lines allowed us to distinguish between the tumor cell- autonomous role and the host tissue role of PPARα. We found that the growth of these isogeneic tumors derived from both cell lines was almost completely suppressed in KO host mice that lacked PPARα, but not in WT animals, p<0.0001 (Figures 1A and 1B). Although tumors derived from MEFs deficient of PPARα were partially suppressed in WT animals (by 41%), indicating a cell-autonomous role of PPAR in tumor growth, a drastic effect in tumor suppression was observed when the host was PPARα deficient both in the case of PPARα(+/+) tumors (87% suppression) as well as PPARα(−/−) tumors (97% suppression) (Figure 1C). These results suggest that the presence of PPARα gene in the host animals is essential for tumor growth.

To examine the role of established tumor murine models we first used WT and KO mice derived from WT (S1)×KO (S4) crossmating. The growth of B16-BL6 tumor was almost completely inhibited in the PPARα KO (S1/S4) host, but was not affected in PPARα WT (S1/S4) animals, p<0.0001 (Figure 1D). This result suggests that presence of the PPARα gene in the host tissue is essential to support tumor growth.

Given that the above results clearly suggest that the status of the PPARα locus in the host affects tumor growth, we next evaluated the growth of three PPARα-positive murine tumor models in PPARα KO (S4) animals, including Lewis lung carcinoma (LLC), metastatic B16-F10/GFP melanoma, and B16-BL6 melanoma, p<0.001 (Figure 1E–G). LLC tumors have been reported to grow aggressively at similar rates in the Sv129, C57BL/6 and Sv129/C57BL/6 strains without evidence of transplantation immunity. This suggests that disparity in either minor or major immunohistocompatibility genes does not affect tumor growth in these models [17] (Figure S1). Macroscopic growth of LLC and B16-F10/GFP tumors was completely suppressed in PPARα KO mice, even when mice were monitored for more than 100 days post implantation (Figure 1E–G). Similarly, tumor metastasis was also suppressed in PPARα KO mice. When B16-F10/GFP melanoma cells and engineered PPARα deficient tumor cells, PPARα (−/−) MEF/RS (see below) were injected via tail vein, 21 out of 21 PPARα wild-type (WT) mice died of lung and/or liver metastasis by day 21. In contrast, the PPARα KO hosts suppressed metastatic growth in lung and liver, reducing the infiltration of the tumor cells from 50–70% of normal organ tissue area in the WT hosts to less than 10% tissue area in PPARα KO animals (Figure 1H). Furthermore, the incidence of metastasis, as measured by the number of histologically identified metastatic foci, was strongly suppressed in PPARα KO mice. The majority of microscopic fields of liver sections in PPARα KO mice revealed only one or two metastases compared to 4–5 foci in livers of WT hosts (Figure 1I). Together these findings support the importance of PPARα expression in host cells for tumor development.

**Figure 2:**
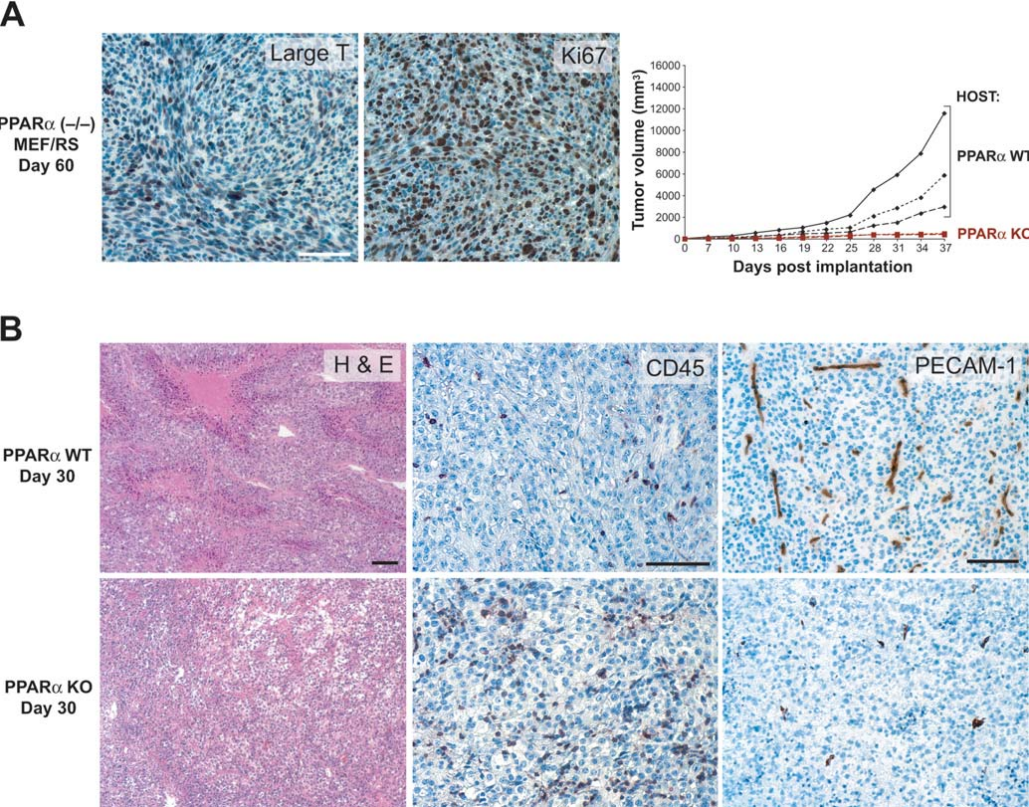
Immunohistological analysis of dormant tumors in PPARα KO mice. The dormant tumors contain viable and proliferating cells, and show decreased microvessel (PECAM1) and increased leukocyte (CD45) staining. (A) Dormant PPARα(−/−)MEF/RS tumors in PPARα KO mice from day 60 post-tumor implantation revealed abundant SV40 large T-antigen staining and proliferation (Ki-67). Dormant PPARα(−/−)MEF/RS tumors on day 60 were implanted as pieces (1 mm^3^) into PPARα WT and KO mice (3 mice in each group). (B) Immunohistochemical analysis of subcutaneous B16-F10/GFP tumors (H&E, CD45/brown color, PECAM-1/brown color) from day 30 post-implantation in PPARα WT mice and KO mice. Scale bars, 100 µm.

The non-growing PPARα(−/−)MEF/RS tumors in PPARα KO mice prompted us to investigate whether these tumors were just a mass of connective tissue or viable dormant microtumors, a state in which tumor cell proliferation is balanced by cell death [18], [19]. Analysis of the small (<2 mm), non-growing lesions at the injection site identified viable PPARα(−/−) MEF/RS large T antigen expressing and proliferating tumor cells (Figure 2A). When re-transplanted to PPARα WT mice, these tumors grew rapidly to over 10,000 mm^3^ (Figure 2A) indicating that PPARα in the host can rescue PPARα −/− tumor cells. Although these findings suggest that the presence of PPARα both in the tumor cells as well as in the host is necessary for unabated tumor growth, they also demonstrate that PPARα in tumor cells is not necessary for tumor cell viability. Conversely, the results underscore the importance of PPARα in the host tissue to sustain tumor growth.

Histological examination revealed a pronounced leukocyte infiltration (based on CD45-positive staining) in the non-necrotic stroma of all tumors grown in PPARα KO mice (Figure 2B). In contrast, PPARα WT animals exhibited the usual leukocytic infiltrate that was limited to necrotic areas (Figure 2B). Moreover, PECAM-1 staining performed to visualize blood capillaries revealed a decreased microvessel density in tumors from PPARα KO hosts when compared to tumors from WT hosts of the same size at day 7 (data not shown), as well as at day 30 post implantation (Figure 2B). Therefore, the absence of PPARα in the stromal tissue of the host appears to have two major consequences: an increase in inflammation and a decrease in tumor angiogenesis.

### Loss of Host PPARα Inhibits Corneal Neovascularization and Permeability

**Figure 3:**
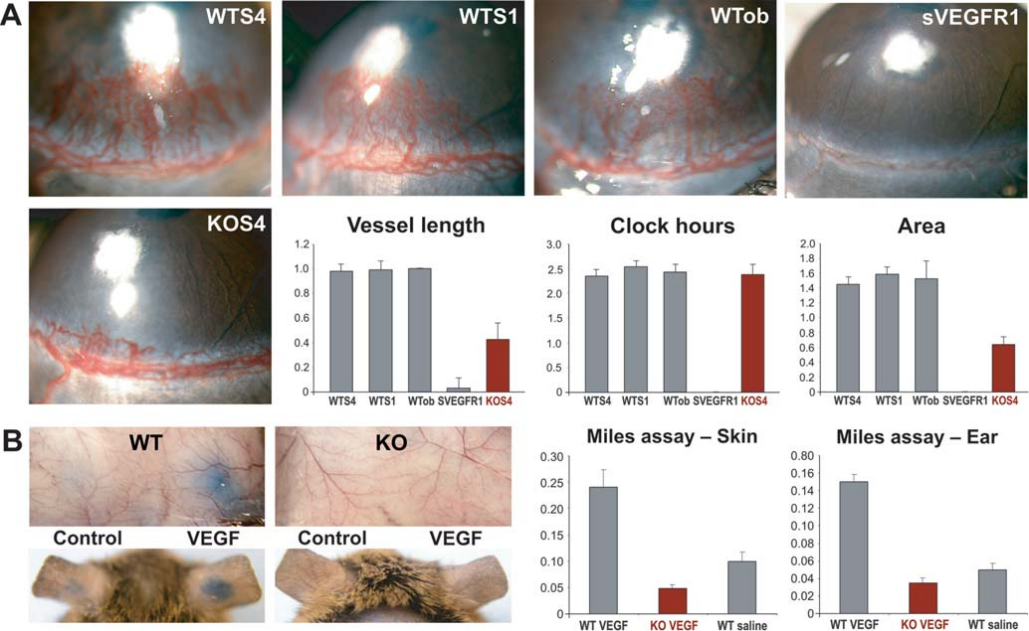
FGF2-induced corneal neovascularization and VEGF-induced vascular permeability are inhibited in PPARα KO mice. (A) FGF-2 (20 ng) stimulates corneal neovascularization in WT 129S4/SvJae strain, WT 129S1/SvIMJ strain and obese WT (129S1/SvJae) mice. Soluble murine VEGFR1 completely inhibits FGF2-induced angiogenesis in WT mouse (sVEGFR1). FGF2-induced corneal neovascularization is potently suppressed in PPARα KO mouse (KOS4). Vessel length, clock hours, and area of neovascularization in PPARα WT and KO mice are represented in bar graphs (average±standard deviation). (B) Evans blue dye leakage in dorsal skin and ears after injection with VEGF or saline in PPARα WT and KO mice (n = 6 mice/group). Spectrophotometric analysis of extravasated Evans blue of skin and ear is represented in bar graph (average±standard deviation).

Decreased microvessel density may reflect direct or indirect antiangiogenic effects caused by the lack of PPARα activity. Because all tumors used here are known to produce the angiogenic cytokine VEGF, we first investigated whether PPARα plays a role in VEGF signaling in the host cells. We employed two different *in vivo* VEGF-activity assays: VEGF-mediated, FGF2-induced corneal neovascularization, and VEGF-induced vascular permeability. Implantation of pellets containing 20 ng of FGF2 into the corneas of mice promotes the extravasation of leukocytes and stimulates VEGF-dependent corneal neovascularization [20], [21]. PPARα KO mice exhibited >50% inhibition of vessel length when compared to WT animals, while the initial sprouting (reflected in clock hours of the neovascularized area) was not affected (Figure 3A). Complete abrogation of angiogenesis in the WT mice in the presence of soluble VEGF-receptor-1 (VEGFR1) confirmed that angiogenesis in these WT animals was mediated by VEGF (Figure 3A), consistent with previous studies [20]. In our second approach, we evaluated whether host PPARα affected VEGF-induced vascular permeability, a standard test of *in vivo* VEGF activity [22], [23]. In response to VEGF, WT mice displayed Evans blue extravasation into the subcutaneous skin and ears (Figure 3B) that was 300–400% greater than that of PPARα KO mice (Figure 3B). Together, these results indicate that host PPARα is indispensable for VEGF-dependent signaling.

### PPARα Deficiency in Bone Marrow Cells Inhibits Tumor Growth

**Figure 4:**
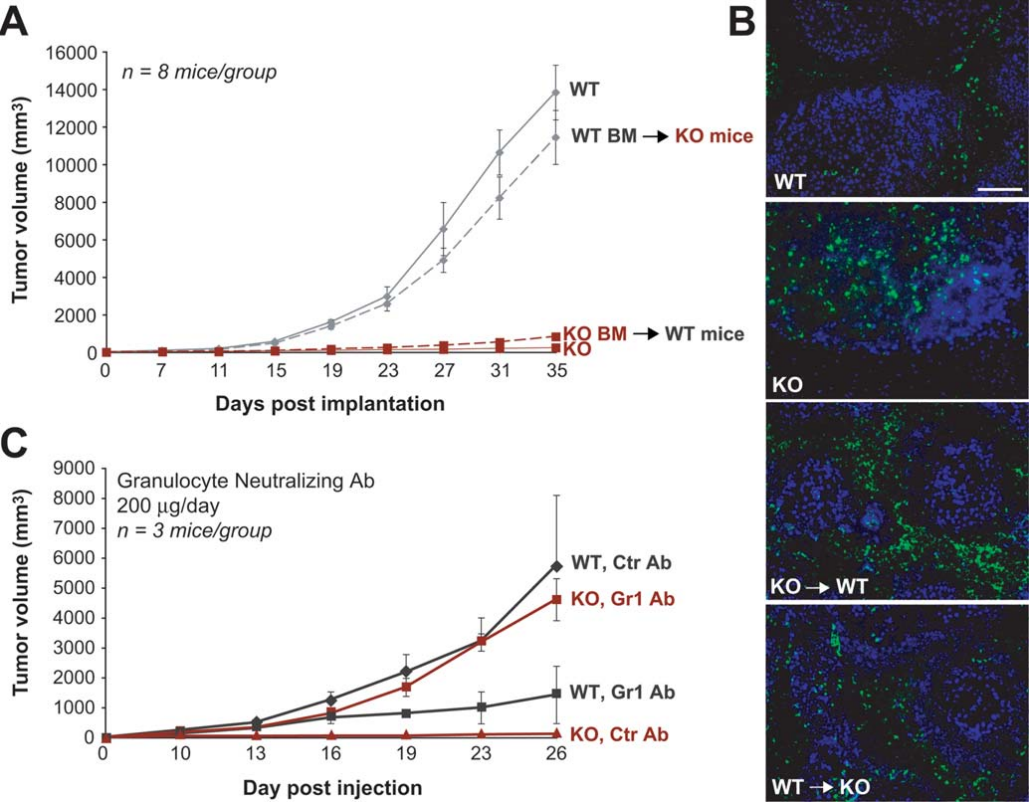
The inhibitory effect of PPARα resides in the hematopoietic compartment. (A) B16-BL6 melanoma growth in WT mice receiving KO bone marrow (KO BM →WT mice) compared to PPARα KO mice receiving WT bone marrow (WT BM →KO mice). WT bone marrow “rescues” tumor growth in PPARα KO mice. (B) Subcutaneous B16-BL6 tumors on day 28 post-implantation show abundant CD45 staining in PPARα WT mice receiving KO bone marrow (KO →WT). In B16-BL6 tumors in KO mice receiving WT bone marrow (WT→KO) CD45 staining (shown in green) was markedly reduced. Hoechst staining of nuclei is blue. Scale bar, 100 µM. (C) Effect of granulocyte depletion using Gr-1 antibody or control antibody (Ctr Ab, IgG2b) on B16-BL6 melanoma growth rate in PPARα KO and WT mice.

Given the observation that the tumor bed of PPARα KO mice exhibited an increased inflammatory response, we performed reciprocal bone marrow transplantations between WT and KO mice to determine whether the hematopoietic compartment of PPARα deficient mice plays a role in the inhibition of tumor growth. Bone marrow cells from WT mice were capable of restoring the “wild-type” tumor growth pattern of B16-BL6 tumors in PPARα deficient hosts (Figure 4A). Conversely, PPARα-deficient bone marrow cells, when transplanted into WT hosts, conferred the tumor-suppressing phenotype of PPARα KO mice, p<0.0001 (Figure 4A). It is important to note that in the bone marrow transplantation protocol used, >90% of the hematopoietic system of the recipient was derived from the donor marrow (Figure S2A); this argues against the possibility that PPARα KO bone marrow cells have a direct, “dominant-negative” effect that overrides a tumor promoting effect of WT bone marrow cells. Instead, the result strongly suggests that the influence of host PPARα on tumor growth is conveyed solely by PPARα activity in bone marrow derived cells, because in these reciprocal transplantation experiments the PPARα status of the transplanted bone marrow cells recapitulates the tumor phenotype of the host. However, it cannot be excluded that the suppressor activity carried by PPARα-deficient bone marrow cells overrides a potential tumor stimulatory contribution of PPARα in other, non-bone marrow derived host cells, such as from the local stroma.

### Depletion of Granulocytes in the PPARα KO Mice Restores Tumor Growth

Immunohistological analysis of B16-BL6 tumors in WT mice transplanted with PPARα-deficient bone marrow cells showed an intense increase in leukocyte staining, mimicking the intratumoral leukocyte profile of tumors grown in PPARα KO mice (Figure 4B). This pronounced leukocyte infiltration in WT mice transplanted with PPARα-deficient bone marrow cells suggests that the presence of PPARα within the inflammatory cells prevents an overt inflammatory response to tumors. Histological and immunohistological analysis of the dormant tumors in PPARα knockout mice revealed that the leukocyte population was predominantly composed of granulocytes, mainly neutrophils (Figure S2B). To corroborate an active role of these PPARα-deficient granulocytes in tumor suppression, we depleted them in the host animals. Flow cytometry analysis confirmed that the granulocyte-specific neutralizing antibody GR1 completely depleted neutrophils (Figure S2C). The anti-granulocyte antibody GR1 restored tumor growth rate in the PPARα KO mice almost completely by day 26 (Figure 4C). In PPARα KO mice that received the control antibody (IgG2b), tumor growth remained inhibited. Conversely, in WT mice the GR1 antibody suppressed tumor growth (Figure 4C vs. 4A), confirming the previous reports that neutrophils are necessary for tumor growth [2], [3]. However, tumor inhibition was even stronger in WT animals whose bone marrow had been replaced with that of PPARα KO mice (Figure 4A) as well as in PPARα deficient hosts (Figure 4A and 4C), again suggesting that not only is PPARα necessary for tumor growth, but that its absence confers a tumor suppressor activity on neutrophils.

### The Inhibitory Role of TSP-1 on Tumor Growth

We next asked why are tumor growth and angiogenesis inhibited by PPARα-deficient leukocytes? Activated inflammatory cells promote angiogenesis, tumor cell proliferation and metastasis through the production of angiogenic mediators, growth factors, chemokines and proteases [2], [24]–[26]. A connection between the positive and negative mediators of the inflammatory response, NF-κB and PPARα, has recently been suggested, because PPARα has been shown to repress NF-κB activity/expression [27]. However, this model disagrees with our result that PPARα-mediated suppression of inflammation is permissive for tumor growth rather than inhibitory. Therefore, our finding suggests that PPARα regulates an aspect of inflammation that is different from that controlled by NF-κB and hence, PPARα modulation of inflammation affects tumor growth independently of NF-κB. While NF-κB exerts its tumor-promoting effect by induction of cytokines, we investigated whether PPARα deficiency suppresses tumor growth by increasing the expression of the matrix protein thrombospondin-1 (TSP-1) which inhibits angiogenesis and stimulates granulocyte migration [28].

**Figure 5:**
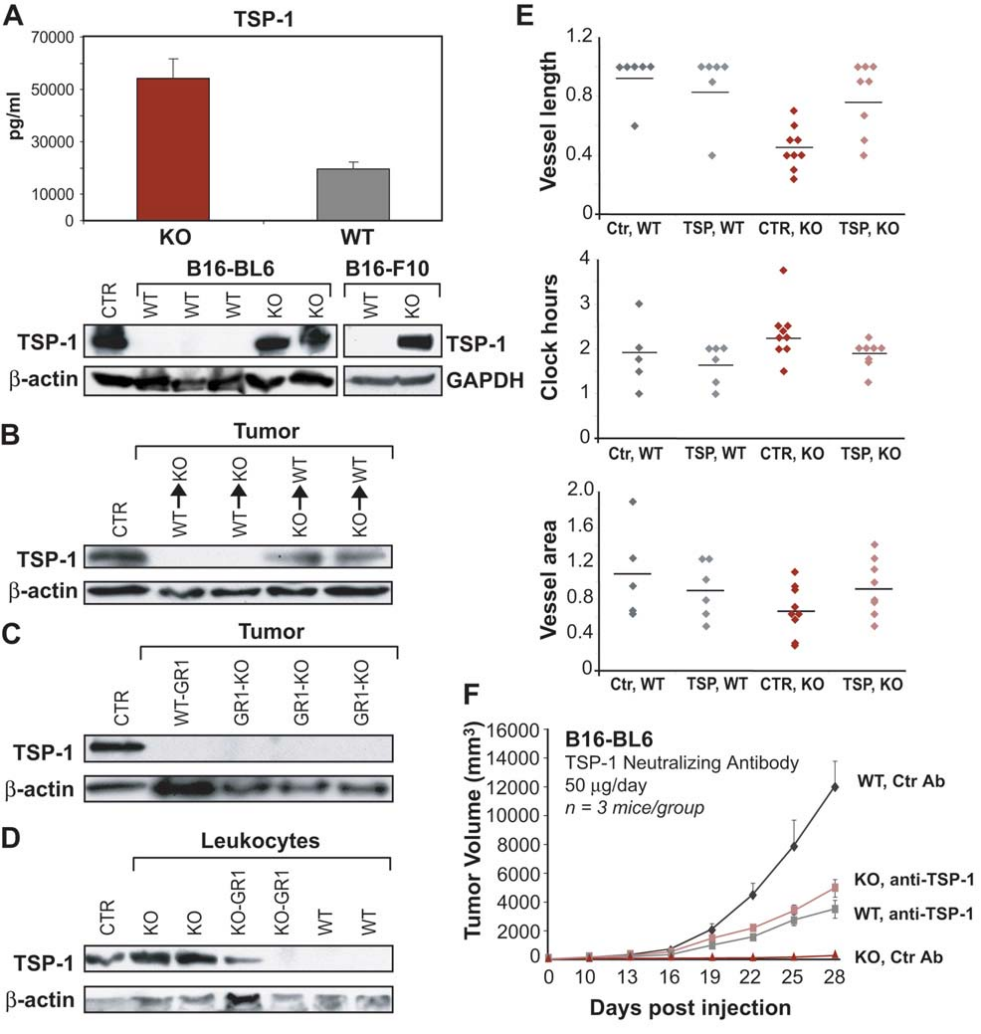
Effects of thrombospondin-1 (TSP-1) on angiogenesis and tumor growth in the PPARα-deficient state. (A) First panel demonstrates TSP-1 levels in plasma of PPARα KO and WT mice (ELISA); second panel shows TSP-1 levels in B16-BL6 and B16-F10/GFP tumor lysates at day 30 grown in PPARα WT and KO mice (western blotting); positive CTR for TSP-1, proliferating HUVECs. (B) Western blot analysis of TSP-1 protein in B16-BL6 tumor lysates from PPARα KO mice receiving WT bone marrow (WT BM→KO), and PPARα WT mice receiving KO bone marrow (KO BM→WT); positive CTR for TSP-1, proliferating HUVECs. (C) TSP-1 protein expression is lost in B16-BL6 tumor lysates from PPARα KO mice depleted of granulocytes (GR-1 antibody); positive CTR for TSP-1, proliferating HUVECs. (D) Western blot analysis of TSP-1 expression from isolated leukocytes from tumor-bearing PPARα KO mice; positive CTR, proliferating HUVECs. Levels of β-actin demonstrate protein loading. (E) Effect of TSP-1 neutralizing antibody and control antibody (IgM) on vessel length (n = 6–9 eyes), clock hours (n = 5–9 eyes) and vessel area (n = 5–9 eyes) in the corneal neovascularization assay. (F) B16-BL6 melanoma growth in KO and WT mice treated with TSP-1 neutralizing or control antibody (IgM).

In fact, TSP-1 was elevated in the plasma and tumor tissue of PPARα KO mice (Figure 5A and Figure S2D). Because TSP-1 can be expressed in several cell types, including tumor cells, endothelial cells and fibroblasts, we next determined the cellular origin for TSP-1 in the tumors of PPARα deficient mice. B16-BL6 and B16-F10/GFP melanomas in PPARα KO mice contained high levels of TSP-1 protein (Figure 5A), despite that these tumor cells do not express TSP-1 [29]. TSP-1 was found in tumors in PPARα WT mice only when the mice received bone marrow from PPARα KO animals. In contrast, little or no TSP-1 was detected in the tumors in PPARα KO mice whose bone marrow cells had been replaced by those from PPARα WT animals (Figure 5B). Moreover, in B16-BL6 tumors from PPARα KO mice treated with GR1 antibody, little or no TSP-1 was detected (Figure 5C). Purified peripheral blood leukocytes from tumor- bearing PPARα deficient mice expressed high levels of TSP-1 while WT leukocytes express very little if any TSP-1 (Figure 5D). Taken together, these findings suggest that in this model system, TSP-1 was produced predominantly by the inflammatory cells, and not by resident stromal cells.

To corroborate the role of TSP-1 in angiogenesis in PPARα deficient animals, we performed the corneal neovascularization assay in the presence of neutralizing anti-TSP-1 antibody. Suppression of vessel length (endothelial cell migration and invasion) in PPARα KO mice was partially reversed by inactivation of TSP-1 function (Figure 5E). There was no effect on the contiguous circumferential zone of the limbal vessel sprouting as measured by clock hours (Figure 5E). In contrast, in the WT mice, corneal neovascularization was not affected by the TSP-1 antibody (Figure 5E).

Provided that neovascularization is a valid marker for tumor angiogenesis, these results are in agreement with the established role of TSP-1 in tumor inhibition [30]. However, we found that the neutralizing TSP-1 antibody did not completely restore tumor growth in PPARα KO mice to the level of that in WT mice, p<0.02 (Figure 5F). This may be either due to the limited access of TSP-1 antibody to the tumor bed or suggests that other endogenous inhibitors of angiogenesis may be involved. In fact, endostatin and IL-12 levels were significantly higher in PPARα KO mice (data not shown). Unexpectedly, we found that in WT animals neutralization of TSP-1 also had an inhibitory (rather than promoting) effect on the tumor, suppressing tumor growth by approximately 71% when compared to control antibody-treated mice, p<0.02 (Figure 5F). This suggests a complex, dualistic role of TSP-1 as a regulator of tumor growth.

## Discussion

In this study we identified the cellular basis for the tumor suppressing phenotype of PPARα deficient mice. Thus, PPARα pathway represents a new link between inflammation, angiogenesis, and tumorigenesis. Absence of PPARα in host granulocytes leads to inhibition of tumor growth, as demonstrated by: (1) transplantation of bone marrow cells from PPARα KO mice to PPARα WT mice and (2) by depletion of granulocytes by the neutralizing antibody, Gr1. Interestingly, PPARα deficient granulocytes carried TSP-1, a protein that inhibits angiogenesis, leukocyte migration and tumor growth. When TSP-1 was depleted by neutralizing antibody in PPARα KO mice, tumor growth was partially reversed.

PPARα is best known as a critical regulator of lipid metabolism and inflammation [31], and is expressed in tissues that catabolize fatty acids such as the liver, as well as in various cell types including smooth muscle cells, monocyte/macrophages, lymphocytes, and endothelial cells [31]. PPARα is the molecular target of the fibrate class of lipid-lowering drugs, which have been widely used for decades in the treatment of dyslipidaemia. Upon activation by PPARα ligands, PPARα heterodimerizes with retinoic acid receptor (RXR) regulating target gene expressions. PPARα ligands act as PPARα agonists. In addition to controlling lipid levels, they also function as potent anti-inflammatory agents in diseases such as atherosclerosis, colitis, and dermatitis [32]–[35]. Accordingly, PPARα KO mice exhibit significant reduction of atherosclerotic lesions, delayed wound healing, and delayed liver regeneration [14], [15], [36], due to overt inflammatory processes. PPARα deficiency also results in a prolonged inflammatory response to lipid mediators [8]. These findings collectively suggest that PPARα has a physiological role in suppressing inflammation [7].

PPARα agonists have been reported to induce liver tumors in rodents, but not in humans [10], [37], [38]. The mechanism for this species difference is still unclear. Accordingly, PPARα KO mice are totally resistant to liver tumors induced by PPARα ligands such as WY-14643 and clofibrate. This indicates that PPARα is required for ligand-induced peroxisome proliferation and hepatocarcinogenesis in rodents in a cell-autonomous manner [9]. It is unclear to what extent this requirement of PPARα for tumor growth is due to tumor cell-autonomous effects or its role in the host compartment of tumors, as shown by our current findings. In our experimental model the suppression of tumor growth in PPARα KO mice is mediated by leukocytes, mainly neutrophils. PPARα deletion is a second example for suppression of tumor growth by ablation of a gene in inflammatory cells; deletion of IKKβ in myeloid cells inhibits epithelial cell tumor growth [26]. However, our model does not exclude a contribution by cell-autonomous tumor promoting effects of PPARα. In fact, we found that deletion of PPARα in the tumor cell itself potentiated the tumor suppressing effect of PPARα-deficiency in the host tissue (Figure 1G and H), in agreement with the earlier reports of the requirement for PPARα in PPARα agonist induced liver tumors [9]. Therefore, PPARα, in addition to NF-κB, may represent another example of an oncogenic protein with a dual role in cancer by controlling essential functions both in cancer cell-autonomous processes as well as processes in the tumor bed, such as inflammation and angiogenesis. Oncogenes and NF-κB have been shown to stimulate tumor cell proliferation and angiogenesis by modifying cytokine expression profiles [25]. Therefore, PPARα does not simply suppress inflammation, acting in opposition to NF-κB, but it does so in a qualitatively different manner in that cellular infiltrates that do not express PPARα, actively suppress rather than stimulate tumor growth.

PPARα-deficient leukocytes produce TSP-1, a potent inducer of leukocyte migration and inhibitor of angiogenesis. Thrombospondin-1 (TSP-1) is a trimeric glycoprotein (450kD) that has several functional domains with different binding affinities. It binds to several cell surface receptors (CD36, integrins αVβ3, α3β1, α4β1, α5β1, heparan sulfate proteoglycans) and also binds calcium and extracellular proteins, such as plasminogen, fibrinogen, fibronectin and urokinase [30], [39]. This multitude of binding partners may explain the diversity of TSP-1 functions: TSP-1 modulates cell adhesion, migration, proliferation and differentiation regulating processes such as inhibition of angiogenesis (through CD36 and β1- integrin) and stimulation of neutrophil migration [28], [40], [41]. TSP-1 is expressed in several cell types in the host: platelets, neutrophils, monocytes, fibroblasts, pericytes, endothelial cells, and tumor cells [42]. Through its role as an activator of TGF-β, it also modulates inflammatory reactions which may contribute to the lethality of TSP-1 KO mice [43]. TSP-1 inhibits tumor growth in mice when overexpressed, putatively via suppression of angiogenesis [40], [44], [45]. However, TSP-1 may also act as a promoter of tumor growth, because anti-TSP-1 receptor antibody inhibited breast tumor growth [46]. Moreover, in vitro TSP-1 has been shown to promote tumor cell invasion and chemotaxis [47]–[49]. In addition, further complicating the picture, in human plasma and tumor stroma the levels of TSP-1 have been correlated with both good and poor cancer prognosis [50]–[56]. This conflicting influence of TSP-1 is recapitulated in our animal model: TSP-1 delivered by leukocytes inhibited tumor growth. However, in the WT animals neutralization of TSP-1 also strongly inhibited tumor growth (Figure 5C). A possible explanation for this apparent paradox is that TSP-1 may have a biphasic effect on angiogenesis and leukocyte migration so that low doses (as found physiologically in WT animals) stimulate and high doses (present in PPARα KO mice) inhibit these processes [57]. Such a “U-shape” dose-effect curve has been reported for many cytokines and bioactive molecules, such as interferon-α, PPARγ ligands and endostatin which all exhibit a biphasic effect on angiogenesis [58]–[62]. Therefore, in WT mice, TSP-1 may operate in the dose-effective window of promoting inflammation which in turn stimulates angiogenesis and tumor growth. In contrast, in PPARα KO mice where TSP-1 is constitutively high, it would act as an inhibitor of tumor growth, perhaps through its antiangiogenic effects. Another possibility, technical rather than biological, is that the activity of TSP-1 is always inhibitory under the conditions studied, but the TSP-1 antibody itself generates the biphasic effect. High levels of TSP-1 in KO mice in the presence of TSP-1 antibodies may promote formation of large antigen - antibody complexes that facilitate TSP-1 clearance, while at low levels, as in WT mice, TSP-1 may be stabilized by the antibody [63].

Given the accumulating findings pointing to the importance for tumor growth of processes in non-cancer host tissues, such as angiogenesis, inflammation and other functions mediated by residual stroma and infiltrating bone marrow cells, our results add a new element to the emerging paradigm that tumor formation is not only a cell-autonomous process. Hence, the action of genes involved in tumor formation must be seen in the broader context of host and tumor [64]. While several pro-inflammatory factors stimulate tumor growth, we report a new molecular link between inflammation and cancer, in that abnormal inflammatory processes can inhibit tumor growth and angiogenesis - thus broadening the spectrum for anticancer therapies that aim at interfering with stromal processes.

## Materials and Methods

### Tumor Xenograft Studies

All the animal studies were reviewed and approved by the animal care and use committee of Children's Hospital Boston. Three to six-month old male PPARα knockout mice (129S4/SvJae), corresponding age-matched WT mice (129S1/SvIMJ, C57BL/6), obese WT mice (129S1/SvIMJ-retired breeders), C3H/HeJ and Balb/cJ mice were obtained from Jackson laboratories (Bar Harbor, ME). Retired WT breeders (35–40 gram) were used to control for weight as PPARα KO mice become obese with age [65]. WT mice (129S4/SvJae) were provided by Dr. John Heymach, Children's Hospital, Boston. PPARα WT and KO littermates were F2 generation. For tumor studies, PPARα negative (−/−) and PPARα positive (+/+) tumors were developed by transforming mouse embryonic fibroblasts (embryonic day 11) isolated from PPARα KO and WT mice, respectively, with SV40 large T-antigen and H-ras (generous gift from Dr. William Hahn). Tumor cells were injected subcutaneously (1×10^6^ cells in 0.1 ml PBS). B16-BL6 melanoma cells were implanted directly from tissue culture; the growth of LLC and B16-F10/GFP tumors was achieved in 129 strains as follows: LLC and B16-F10/GFP cells were first grown in C57BL/6 mice and transplanted as pieces (1 mm^3^) subcutaneously into PPARα WT mice. When tumors were 1000–2000 mm^3^, they were serially passaged from mouse to mouse as 1 mm^3^ pieces and then grown in culture [59]. For experiments, LLC and B16-F10/GFP tumor cells were injected subcutaneously into the 129S PPARα WT and PPARα KO mice either from culture or from mouse to mouse as a cell suspension as described [59]. Tumors were measured every 3–5 days, and the volume was calculated as width^2^×length×0.52. For metastasis studies, 500,000 cells in 0.1 ml PBS were injected via tail vein (n = 15 mice/group). On day 21, when the PPARα WT mice died, all remaining mice were euthanized. Histological sections of livers were quantified for liver metastasis (n = 34–53 fields). For corneal tumor studies, tumor pieces (1 mm^3^) were implanted into the cornea, and the angiogenic response was recorded; photos were taken weekly using a slit-lamp microscope. For granulocyte depletion studies, GR-1 or control antibody (IgG2b) at 300 µg/mouse (Biolegend, San Diego, CA) was administered intraperitoneally two days prior to B16-BL6 melanoma implantation in PPARα WT and PPARα KO mice, and every 3 days post-implantation. Granulocyte depletion was confirmed by flow cytometry using phycoerythrin conjugated Ly-6G (GR-1) antibody (Biolegend, San Diego, CA).

For neutralizing antibody experiments the A4.1 anti-TSP-1 monoclonal antibody (Lab Vision, Fremont, CA) (CSVTCG/CD36) or control antibody (IgM) at 50 µg/mouse were administered intraperitoneally daily to PPARα WT and KO mice in the corneal neovascularization and B16-BL6 melanoma experiments.

### Immunohistochemistry

Tumor samples were processed and immunohistochemical stainings were performed according to standard protocols [59]. For rat anti-mouse PECAM1 (BD Biosciences, San Jose, CA) staining, sections were treated with 40 µg/ml proteinase K (Roche Diagnostics Corp.) for 25 minutes at 37°C. Detection of PECAM1 staining was completed using the tyramide amplification system according to the manufacturer's instructions (PerkinElmer, Boston, MA). For mouse monoclonal thrombospondin-1 (clone A6.1, Lab Vision, Fremont, CA) staining, sections were pretreated with pepsin for 15 minutes at 37°C (Biomeda, Foster City, CA ). For rat anti-mouse CD45 (BD Biosciences, San Jose, CA), and mouse monoclonal NP57 neutrophil elastase (Lab Vision, Fremont, CA) stainings no pretreatments were needed, and stainings were performed using Innogenex IHC kit (San Ramon, CA).

### Angiogenesis Assays

Corneal neovascularization assays were performed. Vessel length was the length of the vessels from the limbal vessel to the pellet. Vessel sprouting was measured as clock hours, the contiguous circumferential zone of the neovascularization, using a 360° reticule (where 30° of arc equals one clock hour). Vessel area was determined using the formula 0.2π×vessel length×clock hours of vessels [66].

For *in vivo* Miles permeability assay, PPARα WT and KO mice received an intravenous injection with 0.5% Evans blue dye (100 µl) retro-orbitally. After ten minutes, the mice were given intradermal injections (50 µl) into the dorsal skin or ear at 2 different sites, consisting of vehicle control or VEGF (50 ng; R&D Systems Inc., Minneapolis, MN). Twenty minutes later the dorsal skin and/or ears were harvested for densitometric analysis to quantify dye leakage. Columns represent mean±standard deviation (n = 6 mice per group; experiments were performed three times).

### Transplantation of Bone Marrow Stem Cells

PPARα WT and KO recipient mice were lethally irradiated with 14 Gy (in a split dose, 4 hours apart) 24 hours before bone marrow transplantation (BMT). Bone marrow cells (1×10^6^) were injected retro-orbitally into recipient mice under isoflurane anesthesia. Neomycin sulfate antibiotic (2 mg/ml) was administered for two weeks post BMT in the drinking water. Mice recovered for a minimum of 2–3 months prior to tumor implantation.

### Western Blot Analysis

For preparation of tumor lysates from PPARα WT and KO mice, B16BL6 tumors were homogenized with protease inhibitor (Roche, Germany). Total protein extracts (50 µg) were analyzed on blots incubated with primary mouse monoclonal TSP-1 (Ab-11, Lab Vision, Fremont, CA) and HRP-conjugated secondary antibodies (Amersham Biosciences Corp. Piscataway, NJ). A positive control for TSP-1 was obtained from exponentially growing HUVECs. For isolation of leukocytes, peripheral blood of PPARα WT and KO mice was obtained by retro-orbital bleeding under isoflurane anesthesia, red cells were cleared by incubating samples for 30 minutes on ice in red blood cell lysis buffer (Sigma-Aldrich, St. Louis, MO). Leukocytes were lysed in 100 µl of a solution consisting of 20 mmol/L imidazole hydrochloride, 100 mmol/L KCl, 1 mmol/L MgCl, 1 mmol/L EGTA, 1% Triton X-100, 10 mmol/L NaF, 1 mmol/L sodium molybdenate, 1 mmol/L EDTA and protease inhibitor cocktail [67].

### TSP-1 ELISA

TSP-1 was measured by ELISA (Cytimmune, Rockville, MD) in blood plasma collected from non-tumor bearing PPARα WT and KO mice. Blood was collected via retro-orbital puncture.

### Statistical Analyses

Statistical ananlyses were performed by Student's *t* test. The results were considered statistically significant for p<0.05.

## Supporting Information

Figure S1 Tumor angiogenesis is inhibited in the cornea of PPARα KO mice. PPARα WT and KO host mice were implanted with tumor pieces (1 mm3) as indicated. (A) Comparison of PPARα(+/+)MEF/RS and PPARα(−/−)MEF/RS in WT mice day 9 and day 16. (B) PPARα(+/+)MEF/RS and PPARα(−/−)MEF/RS in PPARα KO day 9 and day 16. The angiogenic response of PPARα(−/−)MEF/RS in PPARα KO mice regressed by day 16. (C) Lewis Lung Carcinoma (LLC) in PPARα WT and KO, C3H/HeJ and Balb/cJ on day 12. LLC tumors induced tumor angiogenesis independent of host haplotype. Therefore, major histo-incompatibility (MHC) does not prevent tumor-induced neovascularization and tumor growth. In contrast, LLC tumors failed to trigger any angiogenic response in PPARα KO host. (D) B16-BL6 melanoma in PPARα WT and KO on day 16. (E) Histology of B16-BL6 melanoma in the cornea of PPARα WT and KO mice. Scale bars, 500 μm (left) and 100 μm (right) (F) Leukocyte (CD45, brown) staining of LLC tumors in the cornea of PPARα WT and KO mice. Scale bar, 100 µm.
(8.59 MB TIF)

Figure S2 (A) FACS analysis demonstrates % of CD45.1 host cells. In our bone marrow transplantation protocol, >90% of the hematopoietic system of the host was derived from the donor marrow (as proved by using CD45.1 mice as recipients and PPARα KO mice that are CD45.2 as donors). (B) Panleukocyte (CD45, brown) and neutrophil elastase (red) staining in PPARα(−/−)MEF/RS tumors in PPARα WT (day 25) and PPARα KO mice (day 55). Scale bar, 500 µm. (C) FACS analysis demonstrates granulocyte depletion in PPARα KO mice. (D) TSP-1 expression (brown) in B16-F10 (day 30) and PPARα(−/−)MEF/RS (day 60) tumors in PPARα KO and WT mice as determined by immunohistochemical staining. Scale bars, 100μm and 500 µm, respectively.
(5.12 MB TIF)

Text S1 Genetic Background and Transplantation Immunity.
(0.05 MB DOC)
