# Supplementary figures and images for: PPARα Deficiency in Inflammatory Cells Suppresses Tumor Growth
Source: PLoS One. 2007 Feb 28;2(2):e260. doi: 10.1371/journal.pone.0000260 (PMC1800345; doi:10.1371/journal.pone.0000260)

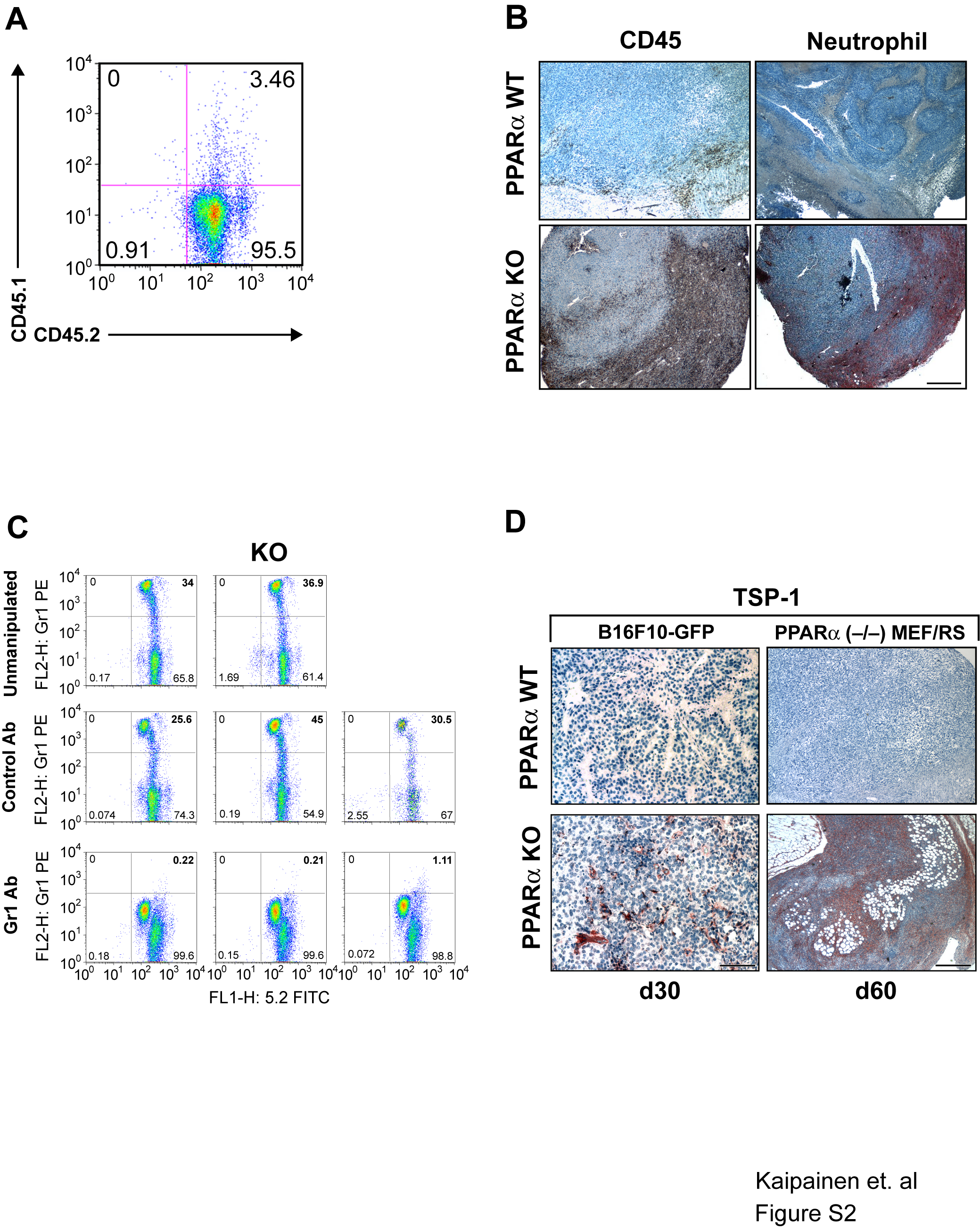

Supplement: Figure S2 — (A) FACS analysis demonstrates % of CD45.1 host cells. In our bone marrow transplantation protocol, >90% of the hematopoietic system of the host was derived from the donor marrow (as proved by using CD45.1 mice as recipients and PPARα KO mice that are CD45.2 as donors). (B) Panleukocyte (CD45, brown) and neutrophil elastase (red) staining in PPARα(−/−)MEF/RS tumors in PPARα WT (day 25) and PPARα KO mice (day 55). Scale bar, 500 µm. (C) FACS analysis demonstrates granulocyte depletion in PPARα KO mice. (D) TSP-1 expression (brown) in B16-F10 (day 30) and PPARα(−/−)MEF/RS (day 60) tumors in PPARα KO and WT mice as determined by immunohistochemical staining. Scale bars, 100μm and 500 µm, respectively. (5.12 MB TIF) [file pone.0000260.s002.tif]
