# Supplementary material for: PPARα Deficiency in Inflammatory Cells Suppresses Tumor Growth
Source: PLoS One. 2007 Feb 28;2(2):e260. doi: 10.1371/journal.pone.0000260 (PMC1800345; doi:10.1371/journal.pone.0000260)
Supplement: Text S1 — Genetic Background and Transplantation Immunity. (0.05 MB DOC) [file pone.0000260.s003.doc]

**SUPPORTING INFORMATION**

**Kaipainen et al.**

**Genetic Background and Transplantation Immunity**

The experiments were performed using PPARα KO mice of the strain 129S4/SvJae(“S4”) whereas the WT control mice were 129S1/SvImJ (”S1”). The S1 line is offered as an "approximate control for the S4 line" by the Jackson Laboratories, and hence, has been used as such in the PPARα field [1], since S4 WT mice are not bred for commercial purpose. Therefore, there is no certified S4 line that could be used as a control for the PPARα KO mice.

The use of S4 as control animals of S1 knockout animals is justified on the following grounds. First, the S1 and S4 “lines” are not nominally different genetic strains. The nomenclature differences refer to two different facilities for maintenance and production of the lines. In 129**S** the *S* states that the mouse strain belongs to the Steel (S) family of 129 strains. S1 and S4 indicate different members of the Steel group of 129 strains.  They have a common origin, but have divergent maintenance histories (See Figure 2 in ref. [2]), reflected in the letters appended to the 129S#/ portion of the strain name. They represent laboratory codes that describe the history of these lines.  As lines are passed from facility to facility, laboratory codes are added to indicate the potential for genetic drift in the isolated colonies: ‘SvJae’ reveals that the 129S4/SvJae line passed through the laboratories of Drs. Leroy Stevens and Rudolf Jaenisch. The 129S1/SvImJ line had a common origin in the Stevens lab and then a line was passed on to the Induced Mutant Resource at The Jackson Lab (Im), and then passed to Jackson laboratories production colonies (J).

According to mouse geneticists at the Jackson laboratory (Jen Merriam, personal communications) there are "no known genetic differences" between these lines based on searches in the Mouse Genome Informatics Polymorphisms database, although the full sequence of these strains is not yet available. However, one article points out differences in 5 out of 86 SSLP markers between the two lines suggesting that there appears to be a formal genetic difference [2]. Therefore we performed a series of experiments to exclude the possibility of differences in transplantation immunity due to these genetic differences outside the PPARα locus that may become manifest in the different murine tumor models:

1. As demonstrated in Figure 1G we used five of the rare WT mice of the S4 substrain (a generous gift from Dr. John Heymach). The data indicate that there is no generic difference in tumor growth in S1 and S4 WT mice for B16-BL6 melanoma, the tumor type we used in the rest of our experiments. We also mated WT (S1) and KO (S4) mice and used the F2 generation litters in the tumor experiments. The tumor growth (B16-BL6) was identical to that of S1 or S4 (Figure 1D).

2. In indirect but clear-cut control experiments, we demonstrate that the suppression of tumor growth in the tumor models used is not affected by the genetic background since the tumors grew even in genetically far more distant mice, even across MHC haplotype: B16-BL6 melanoma (MHC haplotype H2b) was implanted into H2b mice (C57BL/6, PPARα WT 129S1 and 129S4 substrain, and PPARα KO 129S4 substrain). At day 30 after implantation, B16-BL6 melanomas grew at the same rate in PPARα WT substrains and in C57BL/6 mice (Figure 1G). Importantly, despite major MHC disparity, B16-BL6 grew to over 2000 mm3 in the C3H mice (Figure 1G).

3. Since a perfect genetic match between host and tumor was not always available, we generated a tumor line from mouse embryonic fibroblasts derived from the respective prospective host lines (S4 and S1). We showed, as detailed in the main text, that S4-derived transformed MEFs failed to grow normally in S4/PPARα deficient mice, while S1-derived MEF-tumors grew on S1 mice (Figure 1A-C).

4. Finally, PPARα +/+ MEF/RS, and PPARα -/- MEF/RS, and LLC tumors were implanted into the corneas of MHC haplotype mismatched mice. The cornea is avascular and considered to be an “immune-privileged” site with no inherent cell-mediated immunity [3,4]. PPARα +/+ MEF/RS and PPARα -/- MEF/RS grew in the corneas of PPARα WT host. Furthermore, LLC tumors (H2b) grew rapidly in the corneas of C3H (H2k), Balb/cJ (H2d), and PPARα WT (H2b) mice. In contrast, all tumors failed to induce angiogenesis and failed to grow in the PPARα KO host (Figure S1).

There is a theoretical possibility that one of the polymorphisms may directly (rather than via differences in minor transplantation incompatibility which we exclude) account for the dramatic difference in tumor growth that we observe between S1 and S4 animals, while the PPARα locus (presence vs. absence of a gene with implication in host response to tumor!) would play absolutely no role. However, given our collective experience in decades of transgenic mice/mouse work, such a scenario is extremely unlikely. Were this true, this polymorphism would be extremely strong determinants of tumor susceptibility –more than most major oncogenes or tumor suppressor genes and would have gone undetected in all the tumor studies involving 129S mice.

Based on these results and because of the rarity of S4 WT animals, we performed the remaining experiments using the S1 WT strain according to the suggestions by Jackson laboratories.

**Figure Legends**

**Figure S1.** Tumor angiogenesis is inhibited in the cornea of PPARα KO mice. PPARα WT and KO host mice were implanted with tumor pieces (1mm^3^) as indicated. **(A)** Comparison of PPARα(+/+)MEF/RS and PPARα(-/-)MEF/RS in WT mice day 9 and day 16. **(B)** PPARα(+/+)MEF/RS and PPARα(-/-)MEF/RS in PPARα KO day 9 and day 16. The angiogenic response of PPARα(-/-)MEF/RS in PPARα KO mice regressed by day 16. **(C)** Lewis Lung Carcinoma (LLC) in PPARα WT and KO, C3H/HeJ and Balb/cJ on day 12. LLC tumors induced tumor angiogenesis independent of host haplotype. Therefore, major histo-incompatibility (MHC) does not prevent tumor-induced neovascularization and tumor growth. In contrast, LLC tumors failed to trigger any angiogenic response in PPARα KO host. **(D)** B16-BL6 melanoma in PPARα WT and KO on day 16. **(E)** Histology of B16-BL6 melanoma in the cornea of PPARα WT and KO mice. Scale bars, 500 μm (left) and 100 μm (right) **(F)** Leukocyte (CD45, brown) staining of LLC tumors in the cornea of PPARα WT and KO mice. Scale bar, 100 μm.

**Figure S2** **(A)** FACS analysis demonstrates % of CD45.1 host cells. In our bone marrow transplantation protocol, >90% of the hematopoietic system of the host was derived from the donor marrow (as proved by using CD45.1 mice as recipients and PPARα KO mice that are CD45.2 as donors). **(B)** Panleukocyte (CD45, brown) and neutrophil elastase (red) staining in PPARα(-/-)MEF/RS tumors in PPARα WT (day 25) and PPARα KO mice (day 55). Scale bar, 500μm. **(C)** FACS analysis demonstrates granulocyte depletion in PPARα KO mice. **(D)** TSP-1 expression (brown) in B16-F10 (day 30) and PPARα(-/-)MEF/RS (day 60) tumors in PPARα KO and WT mice as determined by immunohistochemical staining. Scale bars, 100μm and 500 μm, respectively.

**References**

1 Fu J, Gaetani S, Oveisi F, Lo Verme J, Serrano A, et al. (2003) Oleylethanolamide regulates feeding and body weight through activation of the nuclear receptor PPAR-alpha. Nature 425: 90-93.

2 Simpson EM, Linder CC, Sargent EE, Davisson MT, Mobraaten LE, et al. (1997) Genetic variation among 129 substrains and its importance for targeted mutagenesis in mice. Nat Genet 16: 19-27.

3 Lyden D, Young AZ, Zagzag D, Yan W, Gerald W, et al. (1999) Id1 and Id3 are required for neurogenesis, angiogenesis and vascularization of tumour xenografts. Nature 401: 670-677.

4 Streilein JW (2003) Ocular immune privilege: therapeutic opportunities from an experiment of nature. Nat Rev Immunol 3: 879-889.
